# Epilepsy: Its Pathology and Treatment

**Published:** 1890-12

**Authors:** 


EPILEPSY: ITS PATHOLOGY AND TREATMENT—Being an Essay to which was Awarded a Prize of Four Thousand Francs by the Academie Royal de Medicine de Belgique, December 31, 1889. By Hobart Amory Hare, M. D., B. Sc., Clinical Professor of the Diseases of Children and Demonstrator of Therapeutics in the University of Pennsylvania, etc. Philadelphia and London. F. A. Davis, publisher: 1890.

The author has displayed commendable zeal and industry in presenting a summary of the available data in regard to the history of Epilepsy, with his views of its pathology and treatment. After careful examination of this little book, in which the theory of the cortical theory of the cerebral origin of the disease is somewhat dogmatically insisted upon, the reader may be inclined to apply to Dr. Hare the language used by him in regard to Dr. Gowers, touching an "inhibition of the motor centres," viz.: "His explanation is of a most hypothetical character and entirely without foundation either physiologically or otherwise."

In respect to treatment, there is absolutely nothing new presented; and the rehearsal of the hackneyed observations upon the use of the bromides as the chief reliance for relief, affords little encouragement to the progressive neurologist or the practitioner in this class of disorders.

It will strike those who are familiar with the failures from medications of various kinds, that the caution against a resort to certain articles is perhaps the most important feature of the essay.

